# Smartphone usage in young adults: cross-sectional associations with physical activity, sleep, social isolation, academic achievement, and anxiety

**DOI:** 10.3389/fpsyt.2025.1722523

**Published:** 2026-01-13

**Authors:** Raghad Alharbi, Alaa Ibrahim, Turki Abualait

**Affiliations:** Department of Physical Therapy, College of Applied Medical Sciences, Imam Abdulrahman Bin Faisal University, Dammam, Saudi Arabia

**Keywords:** academic achievement, anxiety, physical activities, sleep quality, smartphone addiction, social isolation

## Abstract

**Background:**

Smartphones play a vital role in daily life, especially for young adults and university students, providing benefits in communication, learning, and productivity. However, excessive use raises concerns about negative effects on physical and mental health. This study investigates the differences between low-risk and high-risk smartphone addiction users and explores links between overuse and physical activity, sleep quality, social isolation, anxiety, and academic performance among college students in Saudi Arabia.

**Methods:**

This cross-sectional analytic study included 80 undergraduate students aged 18–23 years from Taif University, Saudi Arabia. Participants filled out standardized questionnaires assessing smartphone addiction (SAS-SV), anxiety (GAD-7), social isolation (UCLA-7), and academic performance. Additionally, physical activity and sleep patterns were objectively measured using ActiGraph accelerometers.

**Results:**

Findings revealed that a significant majority (67.5%) of students were classified as high-risk smartphone addiction users. These individuals exhibited significantly elevated levels of social isolation (p=0.016) compared to their peers who utilized smartphones less often. Physical activity differed as well, high-risk users exhibited reduced step counts (p=0.01) and increased sedentary behavior (p=0.008). Sleep assessments also highlighted poorer outcomes in this group, including reduced sleep efficiency (p<0.001) and greater wake time after sleep onset (p<0.001). Among the high-risk users, a strong positive correlation was found between smartphone addiction and anxiety levels (r=0.40, p<0.001).

**Conclusion:**

High-risk smartphone use was linked to social isolation and poorer sleep quality, indicating negative effects on mental and physical health. Anxiety was identified as a statistically significant correlate of smartphone addiction scores in the regression analysis, suggesting emotional dysregulation plays a central role. High-risk smartphone users were also less physically active and more sedentary, though this was not directly tied to addiction scores.

## 1 Introduction

Smartphones have evolved into a crucial component of everyday life, especially for young adults and university students, owing to their extensive application in communication, academic pursuits, entertainment, and social networking. Worldwide, the ownership of smartphones has surged dramatically, with Saudi Arabia exhibiting one of the highest penetration rates globally. In Saudia Arabia, smartphone ownership has increased rapidly from 14.31 million users in 2013 to 33.55 million in 2024, with projections to reach 36.55 million by 2028. Penetration rates rose from 47% in 2013 to 92% in 2024 and are expected to reach 95% by 2028, with ownership at 88%, nearly double the global average of 45% (1, 2). Young adults constitute the most frequent users, which places this demographic at a heightened risk for excessive or problematic smartphone usage. According to the General Authority for Statistics (GASTAT), youth aged 15–34 comprise 36.7% of the Saudi population, with the highest proportions recorded among males aged 20–24 years (27.6%) and females aged 20–29 years (26.2%) (3).

While smartphones provide significant educational and social advantages, an increasing body of evidence indicates that their excessive use may correlate with negative physical, psychological, and behavioral effects. Problematic smartphone usage has progressively been understood through the lens of behavioral addiction, exhibiting characteristics such as compulsive usage, tolerance, withdrawal-like symptoms, and functional impairment. As a result, smartphone addiction has surfaced as a public health issue, especially among university students (4, 5).

Excessive smartphone use has been associated with a wide spectrum of detrimental health and psychosocial outcomes. Physiologically, it contributes to physical inactivity (6), obesity (7), sleep disturbances linked to circadian rhythm disruption (8), and visual strain leading to dry eye disease (9). Prolonged use is also linked to musculoskeletal pain in the neck, shoulders, and hands (10). Psychologically, problematic smartphone use correlates with academic underperformance (11), loneliness and social isolation (12), as well as increased anxiety, depression, and impulsivity, particularly among university students (13). Cognitive impairments involving executive functioning and reward processing further parallel those observed in behavioral addictions (14). Previous research has shown a notable connection between smartphone usage and sleep quality among university students. For instance, Ceylan and Demirdel (15) found that higher smartphone usage correlated with diminished sleep quality, characterized by extended sleep latency and lower sleep efficiency. These results indicate that excessive smartphone usage could adversely affect sleep via behavioral and physiological processes, including procrastination at bedtime and exposure to screens. Collectively, these findings underscore the growing recognition of smartphone overuse as a potential behavioral addiction with significant implications for physical, psychological, and social well-being.

Excessive use of smartphones has been associated with heightened anxiety, social isolation, sleep disturbances, and a decrease in physical activity. Increased exposure to screens may replace daily physical movement and disrupt sleep due to cognitive arousal and disturbances in circadian rhythms (8). Nevertheless, numerous prior studies depend on self-reported data, which constrains the precision of assessments regarding physical activity and sleep. Objective tools such as ActiGraph accelerometers, known for their validity and reliability, allow for precise measurement of physical activity and sleep patterns. However, few studies have applied these devices to assess how smartphone overuse affects these behaviors among university students. In addition, previous research in Saudi Arabia examining the association between smartphone use and academic performance has largely focused on health science students, limiting the generalizability of findings across diverse academic fields. Furthermore, while international studies have consistently reported associations between smartphone addiction, social isolation, and anxiety, local research exploring these psychosocial dimensions remains scarce, despite the unique cultural and societal context in Saudi Arabia.

To address these gaps, the present study incorporates objective assessment of physical activity and sleep using ActiGraph accelerometers and recruits students from multiple academic disciplines, thereby strengthening methodological rigor. This approach allows for a comprehensive examination of the relationships between smartphone addiction and key behavioral and psychosocial domains, including physical activity, sleep quality, social isolation, anxiety, and academic performance, without relying solely on self-reported measures.

## 2 Materials and methods

### 2.1 Study design and setting

This cross-sectional study with analytic and descriptive elements was conducted at Taif University, Western Province of Saudi Arabia, between October 2024 and February 2025.

### 2.2 Sample size

Sample size estimation was performed *a priori* using G*POWER software, based on parameters reported in a previous trial (16). The correlation coefficient (β = 0.076) and effect size (0.275) between smartphone use and sleep quality were applied, with an alpha level of 0.05 and power of 0.80. The minimum required sample size was 80 participants. A *post-hoc* power analysis confirmed adequate power (>0.80) for primary outcomes (sleep efficiency and anxiety). After accounting for a 10% dropout rate, the final estimated sample was 88 students.

### 2.3 Participants and recruitment

**Figure 1:**
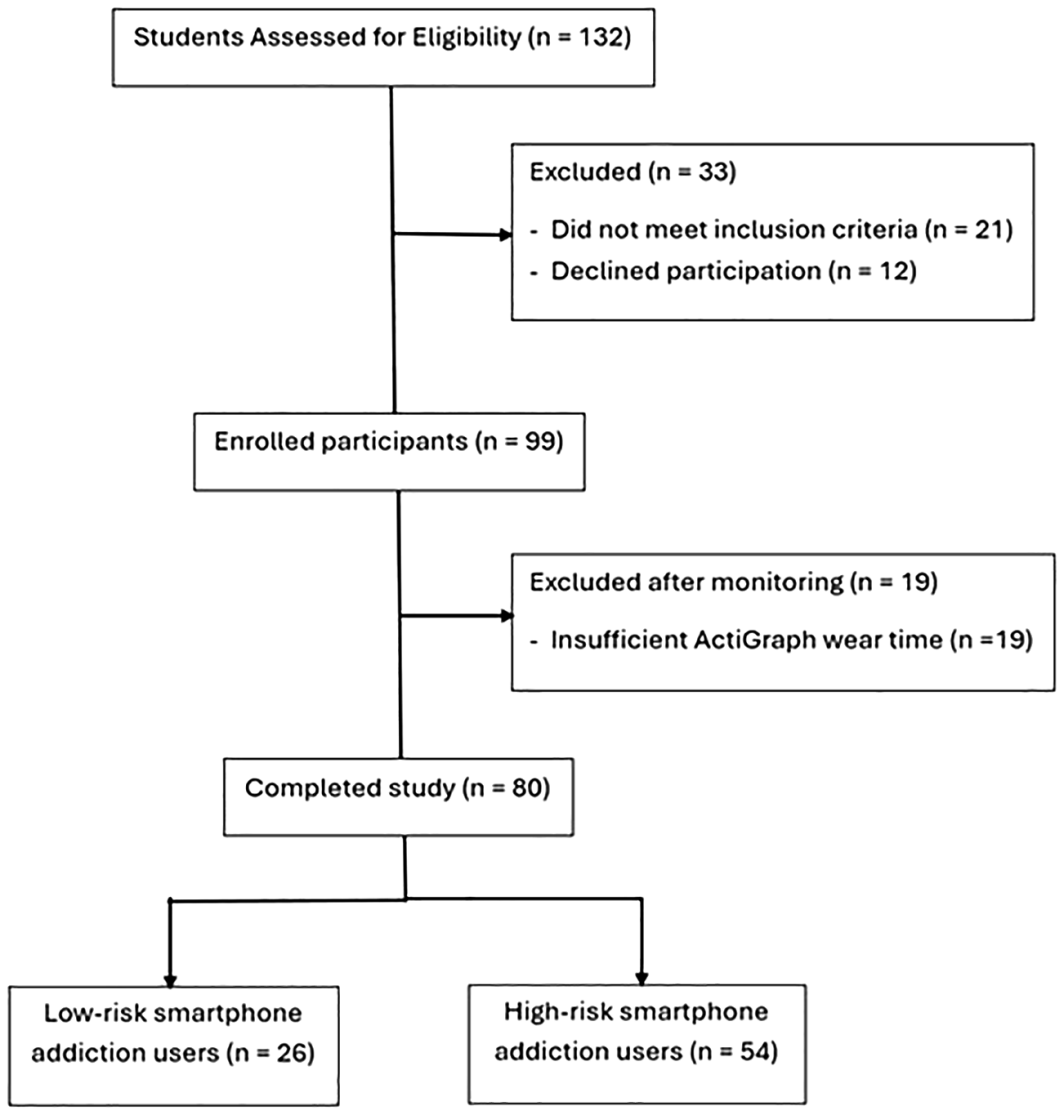
Participants flowchart.

Participants were recruited from various faculties through university email invitations, posters, and in-class announcements. The inclusion criteria consisted of Saudi nationality, an age range of 18 to 23 years, and both male and female participants. The exclusion criteria encompassed psychiatric disorders, neurological conditions, upper limb conditions or amputations that limit smartphone use, sleep disorders, metabolic and endocrine disorders, cardiopulmonary issues, as well as medications recognized for their impact on sleep or cognitive function, in addition to inadequate wear time of the ActiGraph. Students were categorized as low-risk and high-risk smartphone addiction users according to the Smartphone Addiction Scale–Short Version (SAS-SV). Of 132 initially recruited students, 99 were eligible, while 19 were excluded for non-compliance with ActiGraph wear-time requirements (**Figure 1**). Demographic and anthropometric data (sex, age, field of study, academic level, GPA, height, weight, and BMI) were recorded.

### 2.4 Outcome measures

#### 2.4.1 Smartphone addiction

Smartphone use was assessed using the Arabic version of the SAS-SV, which demonstrated high internal consistency (Cronbach’s α = 0.87). The instrument consists of 10 items scored on a six-point Likert scale (1 = strongly disagree to 6 = strongly agree), with total scores ranging from 10 to 60. Higher scores indicate greater severity of smartphone addiction. Cut-off values were 31 for males and 33 for females (17).

#### 2.4.2 Social isolation

Perceived social isolation was assessed using the UCLA Loneliness Scale–Short Form (ULS-7), which evaluates feelings of disconnection (18). Each of the seven items is rated on a four-point Likert scale (1 = never to 4 = always), with scores ranging from 7 to 28. Higher scores reflect greater loneliness. The Arabic version of the ULS-7 demonstrated acceptable reliability among Saudi female students (Cronbach’s α = 0.78) (19).

#### 2.4.3 Anxiety

Anxiety symptoms were evaluated using the Generalized Anxiety Disorder-7 (GAD-7) scale. The original scale has shown excellent reliability (Cronbach’s α = 0.895) (20), while the Arabic version also demonstrated acceptable reliability (Cronbach’s α = 0.763) (21). Total scores range from 0 to 21, with severity classified as: minimal (0–4), mild (5–9), moderate (10–14), and severe (15–21).

#### 2.4.4 Academic achievement

Academic achievement was measured using both last-term GPA and cumulative GPA. Cumulative GPA was categorized according to Taif University’s system: excellent (3.50–4.00), very good (2.75–3.49), good (1.75–2.74), and pass (1.00–1.74).

#### 2.4.5 Physical activity and sleep quality

Physical activity and sleep were assessed using an accelerometer (wGT3X-BT ActiGraph, USA), validated for use in young adults (22). The device (4.6 × 3.3 × 1.5 cm; 19 g) was worn on the non-dominant wrist for four consecutive weekdays, with a minimum of 10 hours/day (23). Devices were removed only during ablution, showering, or swimming. Data were processed with ActiLife software (version 6.13.6). In the analysis, any missing data were addressed through listwise exclusion without the application of imputation. Activity intensity was classified as sedentary (0–99 CPM), light (100–1951 CPM), moderate (1952–5724 CPM), or vigorous (5725–9498 CPM) (24). Output included total step counts and total activity counts.

Sleep outcomes were also extracted, using methods validated in young adults (25–27). Parameters included:

*Sleep efficiency (%) = total minutes asleep ÷ total minutes in bed × 100.*
*Total time in bed (min) = duration from bedtime to wake-up.*
*Total sleep time (min) = cumulative minutes scored as sleep.*
*Wake after sleep onset (min) = total wake time post initial sleep onset.*
*Awakenings (n) = number of discrete wake episodes.*
*Average awakening duration (min) = mean length of wake episodes.*

### 2.5 Statistical analysis

Data analysis was performed using SPSS version 27.0 (SPSS Inc., Chicago, IL). Continuous variables were tested for normality using the Shapiro–Wilk test, histograms, and Q-Q plots. Descriptive statistics were expressed as means ± SD for continuous data and medians or percentages for categorical data. Between-group comparisons were performed using the Mann–Whitney U test. A Bonferroni-adjusted significance level was applied to control multiple comparisons and correlations. Associations were assessed using Pearson’s correlation (continuous variables) and Spearman’s rank correlation (categorical variables). Stepwise multiple linear regression was conducted to identify predictors of smartphone use.

## 3 Results

Based on the Smartphone Addiction Scale–Short Version (SAS-SV), participants were categorized into two groups: 26 students (32.5%) were classified as low-risk smartphone addiction users and 54 students (67.5%) as high-risk smartphone addiction users. The mean (± SD) age, height, weight, and body mass index (BMI) of participants were 20.46 ± 1.32 years, 1.63 ± 0.085 meter (m), 61.76 ± 18.52 kilogram (kg), and 23.03 ± 5.91 kg/m², respectively. The majority of students (90%) were right-hand dominant. The most common academic majors were Nursing (12.5%), Laboratory Sciences (12.5%), and Sport Science (12.5%), followed by Physical Therapy (8.8%), Information Technology (7.5%), and Computer Engineering (7.5%). Academic achievement was concentrated within the “Very Good” GPA category (43.8%), followed by the “Excellent” category (41.3%). The mean social isolation score was 13.08 ± 3.94, and the mean anxiety score was 8.01 ± 5.21 (**Table 1**).

**Table 1:** Demographic, anthropometric, academic, social isolation, and anxiety characteristics of study participants.

Parameters		All participants	Low-risk smartphone addiction users	High-risk smartphone addiction users
Number (N/%)		80(100)	26(32.5)	54(67.5)
Age (Years) (Mean/SD)		20.46(1.32)	20.31(1.38)	20.54(1.30)
Gender (N/%)	Males	32(40)	9(34.6)	23(42.6)
	Females	48(60)	17(65.4)	31(57.4)
Weight (Kg) (Mean/SD)		61.76(18.52)	58.53(15.17)	58.53(15.17)
Height (m) (Mean/SD)		1.63(.085)	1.62(.086)	1.64(.084)
BMI (Mean/SD)		23.03(5.91)	22.05(4.44)	23.49(6.49)
Hand Dominance (N/%)	Right-Handed	72(90.0)	25(96.2)	47(87.0)
	Left-Handed	8(10.0)	1(3.8)	7(13.0)
Specialty (N/%)	Laboratory	10(12.5)	3(11.5)	7(13.0)
	Accounting	2(2.5)	0(0)	2(3.7)
	Physical Therapy	7(8.8)	4(15.4)	3(5.6)
	Information Technology	6(7.5)	3(11.5)	3(5.6)
	Computer Engineering	6(7.5)	2(7.7)	4(7.4)
	Radiology	1(1.3)	0(0)	1(1.9)
	Physics	2(2.5)	0(0)	2(3.7)
	Food Science and Nutrition	5(6.3)	1(3.8)	4(7.4)
	Marketing	1(1.3)	0(0)	1(1.9)
	Business Administration	2(2.5)	0(0)	2(3.7)
	Nursing	10(12.5)	1(3.8)	9(16.7)
	Biotechnology	2(2.5)	2(7.7)	0(0)
	Computer Science	3(3.8)	1(3.8)	2(3.7)
	Executive Secretarial	1(1.3)	0(0)	1(1.9)
	Environmental Protection Technology	2(2.5)	1(3.8)	1(1.9)
	Civil Engineering	4(5.0)	1(3.8)	3(5.6)
	Sport Science	10(12.5)	6(23.1)	4(7.4)
	Biology	3(3.8)	1(3.8)	2(3.7)
	Mechanical Engineering	1(1.3)	0(0)	1(1.9)
	Financial Management	1(1.3)	0(0)	1(1.9)
	Management Information System	1(1.3)	0(0)	1(1.9)
Term GPA (Mean/SD)		3.31(.62)	3.36(.70)	3.29(.59)
Cumulative GPA (Mean/SD)		3.26(.55)	3.27(.55)	3.25(.55)
Cumulative GPA Category (N/%)	Pass	2(2.5)	1(3.8)	1(1.9)
	Good	10(12.5)	2(7.7)	8(14.8)
	Very Good	35(43.8)	12(46.2)	23(42.6)
	Excellent	33(41.3)	11(42.3)	22(40.7)
Social Isolation (Mean/SD)		13.08(3.95)	11.69(3.48)	13.74(4.02)
Anxiety Score (Mean/SD)		8.01(5.212)	6.12(4.49)	8.93(5.33)

GPA, Grade Point Average.

Comparison of demographic, anthropometric, academic, social isolation, and anxiety characteristics between the two groups using the Mann–Whitney test revealed no significant differences except for social isolation and anxiety measures. High-risk users demonstrated significantly higher social isolation scores (p = 0.016) compared to low-risk users. On the other hand, no significant group differences were observed in anxiety scores (p = 0.026) and anxiety category (p = 0.029) levels (**Table 2**).

**Table 2:** Comparing the low-risk and high-risk smartphone addiction users with respect to the demographic, anthropometric, academic, social isolation, and anxiety characteristics (Mann Whitney test).

Parameters		Mean rank	Sig.
Age	Low-risk Users	38.37	0.56
	High-risk Users	41.53	
Gender	Low-risk Users	42.65	0.50
	High-risk Users	39.46	
Weight	Low-risk Users	35.25	0.16
	High-risk Users	43.03	
Height	Low-risk Users	37.73	0.46
	High-risk Users	41.83	
BMI	Low-risk Users	35.81	0.21
	High-risk Users	42.76	
Hand Dominance	Low-risk Users	38.04	0.21
	High-risk Users	41.69	
Specialty	Low-risk Users	41.06	0.88
	High-risk Users	40.23	
Term GPA	Low-risk Users	43.87	0.37
	High-risk Users	38.88	
Cumulative GPA	Low-risk Users	40.25	0.89
	High-risk Users	41.02	
Cumulative GPA Category	Low-risk Users	41.56	0.76
	High-risk Users	39.99	
Social Isolation	Low-risk Users	31.54	0.016
	High-risk Users	44.81	
Anxiety Score	Low-risk Users	32.17	0.026
	High-risk Users	44.51	
Anxiety Category	Low-risk Users	32.75	0.029
	High-risk Users	44.23	

GPA: Grade Point Average; Sig: Bonferroni-adjusted significance level at p = 0.016.

Physical activity outcomes measured by ActiGraph are summarized in **Table 3** low-risk users exhibited significantly higher mean total steps (count) (34,233.85 ± 10,835.87 vs. 27,924.74 ± 8,975.41; p = 0.01), step rate (step/min) (6.13 ± 1.91 vs. 5.11 ± 1.54; p = 0.01), and total time in light activity (minutes) (1,893.58 ± 410.87 vs. 1,490.89 ± 35.35; p < 0.001). Conversely, high-risk users recorded significantly longer total sedentary time (minutes) (3,506.52 ± 509.01 vs. 3,201.62 ± 529.04; p = 0.008). The percentage of total time in light activity (%) was also significantly greater among low-risk users (33.88 ± 7.11 vs. 27.37 ± 6.21; p < 0.001). No significant group differences were observed in axis counts, total activity, activity rate, total time in moderate activities, or percentage of time in moderate activities.

**Table 3:** Comparing the low-risk and high-risk smartphone addiction users with respect to physical activity parameters of the Actigraph (Mann Whitney test).

Parameters		Mean	SD	Mean rank	Sig.
Total Steps (number)	Low-risk Users	34233.85	10835.87	50.62	0.01
	High-risk Users	27924.74	8975.41	35.63	
Step Rate (step/min)	Low-risk Users	6.13	1.91	50.23	0.01
	High-risk Users	5.11	1.54	35.81	
Axis 1 Counts (number of activities per the total wear time)	Low-risk Users	3855110.04	1557907.41	43.38	0.44
	High-risk Users	3711927.22	1343073.60	39.11	
Axis 2 Counts (number of activities per the total wear time)	Low-risk Users	4340006.54	1387806.84	42.35	0.62
	High-risk Users	4150011.15	1443887.86	39.61	
Axis 3 Counts (number of activities per the total wear time)	Low-risk Users	4521723.62	1420125.29	40.81	0.94
	High-risk Users	4517601.24	1410722.70	40.35	
Total Activity (Sum of number of activities in all axes for the total wear time)	Low-risk Users	12716840.19	4317260.86	41.96	0.70
	High-risk Users	12379539.61	4077721.00	39.80	
Activity Rate (Activity/Minute)	Low-risk Users	2281.11	770.66	41.81	0.73
	High-risk Users	2258.19	682.80	39.87	
Total Sedentary Time (minutes)	Low-risk Users	3201.62	529.04	30.58	0.008
	High-risk Users	3506.52	509.01	45.28	
Percentage of Total Sedentary Time (%)	Low-risk Users	57.14	8.10	46.80	>0.001
	High-risk Users	64.19	7.34	27.42	
Total Time in Light Activities (minutes)	Low-risk Users	1893.58	410.87	54.98	>0.001
	High-risk Users	1490.89	353.35	33.53	
Percentage of Total Time in Light Activities (%)	Low-risk Users	33.88	7.11	54.35	>0.001
	High-risk Users	27.37	6.21	33.83	
Total Time in Moderate Activities (minutes)	Low-risk Users	499.77	230.34	44.37	0.30
	High-risk Users	463.54	234.98	38.64	
Percentage of Total Time in Moderate Activities (%)	Low-risk Users	8.97	4.15	43.69	0.39
	High-risk Users	8.43	4.04	38.96	

Sig: Bonferroni-adjusted significance level at p < 0.01.

Sleep parameters are presented in **Table 4** low-risk users showed significantly higher sleep efficiency (%) (91.35 ± 6.53 vs. 80.95 ± 12.69; p < 0.001) and average total sleep time (minutes) (330.24 ± 87.40 vs. 287.77 ± 75.79; p = 0.02). They also had significantly shorter wake time after sleep onset (31.26 ± 24.46 vs. 76.39 ± 67.55; p < 0.001), a lower percentage of wake time after sleep onset (%) (8.65 ± 6.53 vs. 19.05 ± 12.69; p < 0.001), and fewer awakenings per night (count) (13.93 ± 11.82 vs. 33.90 ± 33.12; p = 0.01). No significant differences were observed in total time in bed or mean awakening duration.

**Table 4:** Comparing the low-risk and high-risk smartphone addiction users with respect to sleep parameters of the Actigraph (Mann Whitney test).

Parameters		Mean	SD	Mean Rank	Sig.
Sleep Efficiency (%)	Low-risk Users	91.35	6.53	54.73	>0.001
	High-risk Users	80.95	12.69	33.65	
Average Time in Bed per day (minutes)	Low-risk Users	361.50	90.75	41.17	0.86
	High-risk Users	364.16	112.148	40.18	
Average Sleep Time per day (minutes)	Low-risk Users	330.24	87.40	49.13	0.02
	High-risk Users	287.77	75.79	36.34	
Average Wake Time After Sleep Onset per day (minutes)	Low-risk Users	31.26	24.46	28.54	>0.001
	High-risk Users	76.39	67.56	46.26	
Percentage of Wake Time After Sleep Onset (%)	Low-risk Users	8.65	6.53	26.27	>0.001
	High-risk Users	19.05	12.69	47.35	
Average Number of Awakenings per day (number)	Low-risk Users	13.93	11.82	30.31	0.01
	High-risk Users	33.90	33.12	45.41	
Average wakening time of one Awakening (minutes)	Low-risk Users	2.38	0.44	41.38	0.81
	High-risk Users	2.40	0.51	40.07	

Sig: Bonferroni-adjusted significance level at p < 0.01.

Spearman correlation analyses (**Table 5**) showed that among low-risk users, smartphone addiction scores correlated positively with both anxiety score (r = 0.44, p = 0.02) and anxiety category (r = 0.47, p = 0.02). Among high-risk users, smartphone addiction scores were similarly correlated with anxiety score (r = 0.40, p < 0.001) and anxiety category (r = 0.33, p = 0.02). No significant correlations were found with demographic, anthropometric, academic, or social isolation variables in either group.

**Table 5:** Correlations between smartphone addiction score and demographic, anthropometric, academic, social isolation, and anxiety characteristics in study participants.

Spearman correlations													
Study participants		Age	Gender	Weight	Height	BMI	Hand dominance	Term GPA	Cumulative GPA	Cumulative GPA category	Anxiety score	Anxiety category	Social isolation
Low-risk Users	r	-0.08	0.18	-0.21	-0.18	-0.13	-0.17	-0.06	-0.17	-0.15	0.44	0.47	0.20
	p	0.72	0.38	0.30	0.38	0.54	0.40	0.76	0.40	0.48	0.02	0.02	0.33
High-risk Users	r	-0.25	-0.13	0.01	0.25	-0.08	-0.13	-0.23	-0.22	-0.20	0.40	0.33	0.26
	p	0.07	0.36	0.97	0.07	0.57	0.34	0.10	0.11	0.15	>0.001	0.02	0.06

GPA: Grade Point Average; r: Correlation Coefficient; p: Bonferroni-adjusted significance level at p < 0.016.

No significant correlations were identified between smartphone addiction scores and physical activity parameters in either group (**Table 6**). Correlation coefficients ranged from −0.09 to 0.12, with all p-values >0.05, indicating weak and nonsignificant associations.

**Table 6:** Correlations between smartphone addiction score and physical activity parameters of the Actigraph in study participants.

Spearman correlations											
Study participants		Total steps	Step rate	Total activity	Activity rate	Total sedentary time	Percentage of total sedentary time	Total time in light activities	Percentage of total time in light activities	Total time in moderate activities	Percentage of total time in moderate activities
Low-risk Users	r	-0.05	-0.06	0.02	-0.01	-0.03	-0.04	0.04	0.10	0.02	0.04
	p	0.81	0.76	0.93	0.95	0.89	0.84	0.86	0.63	0.93	0.86
High-risk Users	r	-0.01	-0.08	0.03	-0.01	0.09	-0.02	0.12	0.08	-0.04	-0.08
	p	0.93	0.55	0.82	0.97	0.54	0.92	0.39	0.59	0.76	0.59

r: Correlation Coefficient; p: Bonferroni-adjusted significance level at p < 0.01.

As shown in **Table 7**, smartphone addiction scores were not significantly correlated with sleep parameters in low-risk users. However, among high-risk users, significant associations were observed: a negative correlation with sleep efficiency (r = −0.27, p = 0.05) and a positive correlation with percentage of wake time after sleep onset (r = 0.27, p = 0.05).

**Table 7:** Correlations between smartphone addiction score and sleep parameters of the Actigraph in study participants.

Spearman correlations								
Study participants		Sleep efficiency	Average time in bed per day	Average sleep time per day	Average wake time after sleep onset per day	Percentage of wake time after sleep onset	Average number of awakenings per day	Average wakening time of one awakening
Low-risk Users	r	0.08	-0.02	0.003	-0.10	-0.08	-0.12	0.30
	p	0.69	0.94	0.99	0.63	0.69	0.55	0.14
High-risk Users	r	-0.27	0.08	-0.01	0.25	0.27	0.25	0.06
	p	0.05	0.55	0.95	0.07	0.05	0.07	0.67

r: Correlation Coefficient; p: Bonferroni-adjusted significance level at p < 0.01.

Finally, multiple linear regression analysis among high-risk users (**Table 8**) showed that anxiety score, anxiety category, and percentage of wake time after sleep onset explained 20% of the variance in smartphone addiction scores (R² = 0.20, p = 0.01). Of these, anxiety score emerged as a significant independent predictor (B = 0.94, p = 0.04).

**Table 8:** Prediction of smartphone addiction score in high-risk users (Multiple Linear Regression).

Multiple linear regression		
Smartphone addiction score		
Predictors	B	Sig
Anxiety Score	0.94	0.04
Anxiety Category	-0.58	0.20
Sleep Efficiency	Excluded for collinearity	
Percentage of Wake Time After Sleep Onset	0.15	0.23
Model Summary		
R Square (Sig)	0.20 (0.01)	

B: Standardized Coefficients Beta.

## 4 Discussion

### 4.1 Smartphone addiction prevalence

The objective of this cross-sectional study was comparing physical activity, sleep quality, social isolation, anxiety levels, and academic performance between low-risk and high-risk users and to detect any possible association between high-risk smartphone use by college students and their physical activity, sleep quality, social isolation, anxiety, and academic performance. A significant proportion of the participants (67.5%) were classified as high-risk smartphone addiction users, consistent with previous findings highlighting the widespread prevalence of high smartphone use among college students (28). This elevated usage may be attributed to academic demands, social communication, and entertainment, all of which are increasingly mediated through smartphones (29). The high rate of the observed excessive use warrants concern, especially considering its potential impact on mental health and academic functioning. The study by Venkatesh et al. (30) showed that 71% of the dental students in Saudi Arabia were smartphone-addicted, which was in line with our research findings. However, it is higher than the global prevalence (26.99%), as well as the prevalence in other countries, reported lower rates of smartphone addiction among young people in China (49.5%) (31).Turkey (34.8%) (32), and United Kingdom (38.9%) (33).

### 4.2 Smartphone addiction, demographic, anthropometric, and academic characteristics

Results indicated no statistically significant differences between the groups in terms of age, gender, anthropometric measures (weight, height, BMI), hand dominance, academic specialty, or academic performance (term GPA, cumulative GPA, GPA category). These findings suggest that high-risk smartphone use may be independent of demographic factors, a conclusion echoed by earlier studies that also reported no consistent demographic predictors of smartphone addiction (34).

Interestingly, academic performance, measured by both term and cumulative GPA, did not differ significantly between low-risk and high-risk users. This finding contrasts with some studies that have suggested negative academic consequences of smartphone overuse (34–36). One possible explanation is that students may engage in compensatory behaviors (e.g., multitasking or late-night studying) or may be using smartphones as academic tools, thus minimizing the impact on grades. However, academic performance alone may not fully capture the potential functional impairments associated with smartphone addiction, especially in terms of emotional and social well-being.

### 4.3 Smartphone addiction and psychological characteristics

Significant differences were observed in key psychological outcomes. High-risk users reported higher levels of social isolation (p = 0.016), which is consistent with previous research highlighting the paradoxical effect of smartphone use: while devices are intended to facilitate connection, overuse, particularly on social media platforms, can lead to perceived disconnection and loneliness (37–39). The immersive nature of smartphones may displace face-to-face interactions, weakening real-world social bonds and fostering feelings of exclusion or isolation.

### 4.4 Smartphone addiction and physical activities

The present study investigated differences in objectively measured physical activity parameters between low-risk and high-risk users using ActiGraph accelerometry. The findings revealed significant reductions in physical activity levels and increased sedentary behavior among high-risk users compared to their low-risk users’ peers.

Specifically, low-risk users demonstrated significantly higher total steps, step rate, and total time spent on light physical activity, while high-risk users recorded higher total and percentage sedentary time. These results are consistent with previous research showing a negative association between high-risk smartphone use and physical activity levels. For example, Jeong et al. (40) reported that problematic smartphone use was associated with increased sedentary time and decreased moderate-to-vigorous physical activity (MVPA) among university students. The findings observed align with the behavioral displacement hypothesis, which posits that increased screen-related activities correlate with diminished participation in active endeavors (41, 42).

The increased sedentary behavior observed in high-risk users is particularly concerning, as prolonged sedentary time has been linked to a range of adverse health outcomes, including obesity, cardiovascular disease, and mental health disorders. Participation in smartphone-related activities, including gaming, social media exploration, and video streaming, was linked to increased sedentary behavior, indicating a connection between smartphone usage and sedentary lifestyles (43, 44).

Furthermore, the lack of significant differences in moderate-intensity activity between groups suggests that the main behavioral differences lie in low-intensity activities and sedentary time rather than structured or vigorous exercise. This supports the notion that high-risk smartphone use is associated with differences in non-exercise activity thermogenesis (NEAT)-related behaviors, particularly in light-intensity activity and sedentary time that make up the majority of daily physical activity in non-athletes (43).

High-risk smartphone use is linked to decreased physical activity; conversely, excessively high levels of physical activity can negatively affect academic performance by reducing study time. This highlights the importance of determining the ideal daily activity levels for university students. While specific thresholds for accelerometer use have not yet been established, the World Health Organization (45) advises that adults aged 18 to 64 should engage in a minimum of 150 minutes of moderate-to-vigorous physical activity each week, or a suitable mix of activity intensities, to gain health benefits.

Importantly, these findings underscore the role of smartphones in shaping daily movement patterns among college students. While smartphones can be leveraged for health-promoting purposes (e.g., fitness tracking apps or step reminders), their overuse appears to displace incidental physical activity and promote a sedentary lifestyle. Given that even light activity has important health benefits, especially when replacing sedentary time (42), the results highlight a need for interventions aimed at reducing passive smartphone use and encouraging movement throughout the day.

### 4.5 Smartphone addiction and sleep quality

This study investigated differences in sleep quality between low-risk and high-risk users using objective sleep metrics. The results revealed that high-risk smartphone use is significantly associated with impaired sleep quality. Specifically, low-risk users demonstrated higher sleep efficiency, longer total sleep duration, and lower levels of sleep fragmentation compared to high-risk users. These findings align with an expanding body of literature linking problematic smartphone use to poor sleep outcomes among university students.

The significantly lower sleep efficiency among high-risk users (80.95%) compared to low-risk users (91.35%) reflects a disrupted sleep pattern, consistent with a previous study for Lee et al. (46). Poor sleep efficiency indicates that individuals spend more time in bed awake rather than asleep, which has been associated with higher stress levels and diminished cognitive performance (47).

Moreover, the shorter average sleep duration in high-risk users (approximately 288 minutes per night) supports earlier findings that screen exposure, particularly in the pre-sleep period, can delay sleep onset and reduce total sleep time (48, 49). Prior studies have indicated that exposure to blue light emitted by smartphones is linked to changes in melatonin production and circadian rhythms, potentially contributing to challenges in both the onset and maintenance of sleep (50).

The increased wake time after sleep onset (WASO) and a greater number of awakenings among high-risk users suggest heightened sleep fragmentation, which can reduce the restorative quality of sleep (51). These sleep disruptions could be associated with elevated levels of psychological arousal and anxiety, which were found to be more common among individuals exhibiting higher scores of smartphone addiction (52).

Interestingly, no significant differences were found in the average time in bed or in the duration of individual awakenings. This implies that the quantity of time allocated for sleep may be similar between groups, but the quality and continuity of sleep are substantially worse in high-risk smartphone users. This distinction highlights the importance of assessing not just sleep quantity but also sleep architecture and fragmentation when studying the effects of digital behavior.

Taken together, the results of this study reinforce concerns that high-risk smartphone use correlates with diminished sleep quality through multiple pathways, including circadian disruption, increased cognitive and emotional arousal, and habitual nocturnal engagement. Poor sleep, in turn, may have cascading effects on physical health, mental well-being, and academic functioning (53, 54).

### 4.6 Association with smartphone addiction

The current study explored the associations between smartphone addiction and various psychological, academic, anthropometric, physical activity, and sleep-related parameters among low-risk and high-risk users. A major finding was the consistent, statistically significant positive correlation between smartphone addiction scores and anxiety levels in both user groups. This aligns with prior research indicating that elevated anxiety is a common psychological correlate of problematic smartphone use (55, 56). In the current study, this relationship persisted across low-risk users (r = 0.44, p = 0.02) and high-risk users (r = 0.40, p > 0.001), reinforcing the role of anxiety as a robust predictor of maladaptive smartphone behaviors.

Further, the positive correlation between smartphone addiction and categorical anxiety levels (r = 0.47 and r = 0.33 for low-risk and high-risk users, respectively) underscores the clinical relevance of this association. High-risk smartphone use may serve as a maladaptive coping mechanism for managing anxiety, or conversely, increased screen time may exacerbate anxiety symptoms through disrupted sleep, information overload, and reduced face-to-face interactions (57, 58).

Interestingly, no significant associations were observed between smartphone addiction and demographic or academic variables, including age, gender, GPA, or social isolation in either group. These findings are consistent with some previous studies that found no consistent demographic predictors of smartphone addiction (59, 60), suggesting that psychological and behavioral factors may play a more central role.

Contrary to expectations, physical activity levels, objectively measured via ActiGraph, were not significantly correlated with smartphone addiction scores across any of the measured parameters in both groups. These findings diverge from prior reports suggesting that excessive screen time may displace time spent on physical activities (61, 62). The absence of a significant relationship may be attributed to compensatory behaviors among students, or the possibility that smartphone use does not uniformly affect all types of physical activity (e.g., active commuting versus structured exercise). Additionally, the homogeneity of physical activity levels within the sample may have attenuated the detection of significant associations.

Ceylan and Demirdel (15) also found a notable link between smartphone usage and levels of physical activity, suggesting that higher smartphone use could change daily activity habits. These results reinforce our finding of diminished objective physical activity in individuals who excessively use smartphones and emphasize the importance of establishing ideal physical activity levels instead of presuming a straightforward protective effect.

Sleep parameters, on the other hand, showed nuanced associations. While low-risk users exhibited no significant correlations between smartphone addiction and sleep characteristics, high-risk users demonstrated a significant negative correlation between smartphone addiction and sleep efficiency (r = –0.27, p = 0.05), as well as a significant positive correlation with the percentage of wake time after sleep onset (r = 0.27, p = 0.05). These findings are congruent with previous literature linking problematic smartphone use to poor sleep quality, likely due to blue light exposure, cognitive arousal, or bedtime procrastination (63).The impact was more prominent among high-risk users, suggesting that higher levels of usage are more likely to disrupt circadian patterns and sleep continuity.

The current results align with the observations made by Ceylan and Demirdel (15), which indicated that higher smartphone usage correlates with diminished sleep quality in university students. Collectively, these results imply that both subjective and objective assessments of sleep are negatively impacted by excessive smartphone usage.

Supporting this, the multiple linear regression model identified anxiety score as a significant independent predictor of smartphone addiction in high-risk users (B = 0.94, p = 0.04), accounting for 20% of the variance in addiction scores (R² = 0.20, p = 0.01). Although sleep efficiency was excluded from the model due to collinearity, its correlation with addiction further highlights its intertwined role in this behavioral pattern. The overall model underscores the complex interplay between emotional and behavioral variables in driving excessive smartphone use.

### 4.7 Limitations

While this study provides valuable insights, several limitations should be acknowledged. First, the cross-sectional design precludes any inference of causality or directionality among the examined variables. Second, the relatively small sample size may limit the statistical power and robustness of the findings. Third, many independent variables were based on self-reported data, which introduces the possibility of recall bias. Fourth, the use of convenience sampling limits the representativeness of the sample, restricting the generalizability of the results to broader or similar populations. Fifth, physical activity and sleep quality were measured over only four days; a seven-day monitoring period would have yielded more comprehensive and reliable data. sixth, the use of the ULS-7 scale, validated only in female populations, limits the applicability of the results across genders. Finally, the duration of daily smartphone usage was not objectively documented through device-based logs, and the evaluation of blue light filtering features during both daytime and nighttime smartphone usage was not conducted. These elements could potentially affect sleep quality via mechanisms such as heightened screen exposure and disruption of circadian rhythms. Future research is recommended to include objective data on smartphone usage collected from integrated device tracking systems, as well as to assess the impact of blue light filtering or night mode functionalities.

## 5 Conclusions

This study explored the multifaceted impacts of high-risk smartphone use among college students, particularly its associations with physical activity, sleep quality, social isolation, anxiety, and academic performance. The results revealed that a substantial majority of participants (67.5%) exhibited high-risk smartphone use, reflecting a growing global trend. While no significant differences were observed in demographic or academic characteristics between low-risk and high-risk users, marked disparities emerged in psychological and behavioral domains.

High-risk smartphone use was significantly linked to higher levels social isolation supporting the growing body of evidence that suggests problematic smartphone behaviors were associated with indicators of poorer mental well-being, particularly higher levels of social isolation. Notably, anxiety emerged as a consistent and independent predictor of smartphone addiction, suggesting that emotional dysregulation may be a central factor driving maladaptive usage patterns.

Objective measures revealed that high-risk users engaged in significantly less physical activity and more sedentary behavior. However, no direct correlation was found between smartphone addiction scores and activity levels, indicating that overuse may affect movement patterns in complex or indirect ways. Sleep quality, on the other hand, was notably impaired in high-risk users, with correlations showing poorer sleep efficiency and greater sleep fragmentation. The results of this study suggest that an increased risk of smartphone addiction correlates with reduced levels of physical activity and diminished sleep quality, both of which are crucial for maintaining mental and physical well-being.

## Data Availability

The original contributions presented in the study are included in the article/supplementary material. Further inquiries can be directed to the corresponding author/s.
